# Solid-surface vitrification is an appropriate and convenient method for cryopreservation of isolated rat follicles

**DOI:** 10.1186/1477-7827-8-42

**Published:** 2010-05-11

**Authors:** Weijie Xing, Canquan Zhou, Jiang Bian, Markus Montag, Yanwen Xu, Yubin Li, Tao Li

**Affiliations:** 1 Reproductive Medicine Center, the First Affiliated Hospital of Sun Yat-sen University, Guangzhou, China; 2 Department of Gynecological Endocrinology and Reproductive Medicine, University Clinics Bonn, Sigmund-Freud-Str. 25, 53105 Bonn, Germany

## Abstract

**Background:**

Cryopreservation of isolated follicles may be a potential option to restore fertility in young women with cancer, because it can prevent the risks of cancer transmission. Several freezing protocols are available, including slow-rate freezing, open-pulled straws vitrification (OPS) and solid-surface vitrification (SSV, a new freezing technique). The purpose of our study was to investigate the effects of these freezing procedures on viability, ultrastructure and developmental capacity of isolated rat follicles.

**Methods:**

Isolated follicles from female Sprague-Dawley rats were randomly assigned to SSV, OPS and slow-rate freezing groups for cryopreservation. Follicle viability assessment and ultrastructural examination were performed after thawing. In order to study the developmental capacity of thawed follicles, we performed *in vitro *culture with a three-dimensional (3D) system by alginate hydrogels.

**Results:**

Our results showed that the totally viable rate of follicles vitrified by SSV (64.76%) was slightly higher than that of the OPS group (62.38%) and significantly higher than that of the slow-rate freezing group (52.65%; *P *< 0.05). The ultrastructural examination revealed that morphological alterations were relatively low in the SSV group compared to the OPS and slow-rate freezing groups. After *in vitro *culture within a 3D system using alginate hydrogels, we found the highest increase (28.90 ± 2.21 μm) in follicle diameter in follicles from the SSV group. The estradiol level in the SSV group was significantly higher than those in the OPS and slow-rate freezing groups at the end of a 72-hr culture period (*P *< 0.05).

**Conclusions:**

Our results suggest that the SSV method is an appropriate and convenient method for cryopreservation of isolated rat follicles compared with the conventional slow-rate freezing method and the OPS method.

## Background

Ovarian cortical tissue contains high numbers of primordial and primary follicles. Hence cryopreservation of ovarian cortex has become a potential option to restore fertility in young women with cancer [1]. However, many studies have shown the risks of cancer transmission following transplantation of ovarian tissues especially in cases of blood-borne cancer. Oktay et al. [2] reported the first successful isolation of primordial follicles from human ovaries by a gentle enzymatic technique using collagenase in combination with manual dissection. As a result, an optimized protocol with a mixture of purified collagenase for digestion has been developed. This method yields good-quality isolated human follicles, which is an absolute prerequisite for further successful processing of these follicles, either for culture or transplantation [3].

Several freezing protocols are available, including slow-rate freezing, open-pulled straws vitrification (OPS) and solid-surface vitrification (SSV, a new freezing technique). Vitrification involves rapid cooling and warming rates in the presence of very high concentration of cryoprotectants to prevents intercellular ice-crystal formation and chilling injury [4]. SSV, a new freezing technique, has been successfully applied to preserve oocytes [5-7] and ovarian tissues [8]. SSV uses a metal surface, which is pre-cooled to -180°C by partial immersion into liquid nitrogen (LN_2_) and serves as a template to cool microdrops of vitrification solution containing embryos or oocytes [9]. SSV provides enough space for tissue, maximizes cooling rates, and avoids the generation of gas phase of LN_2 _bubbles [10].

The aim of our study was to investigate the effects of the three freezing methods (SSV, OPS and slow-rate freezing) on viability, ultrastructure and developmental capacity of isolated rat follicles.

## Methods

### Animals

Three-week-old female Sprague-Dawley rats were purchased from the Animal Center of Chinese Academy of Sciences (Shanghai, China) and bred under temperature controlled conditions with a 12-hr light-dark cycle. Ovaries were collected from these rats for follicle isolation.

### Follicle isolation

Follicles were isolated as previously described [3]. Briefly, each biopsy was cut into uniformly sized pieces of 0.5 × 0.5 × 1 mm by a tissue chopper. Subsequently, the fragments were digested with 0.04 mg/mL Liberase Blendzyme 3 (Roche, Indianapolis, USA) in 15 mL conical flasks, which were incubated in a water bath at 37°C for 75 min. The ovarian digest was mixed with a pipette to mechanically disrupt the digested tissue every 15 min. Digestion was terminated by adding an equal volume of phosphate-buffered saline (PBS; Gibco, USA) supplemented with 10% fetal bovine serum (FBS; Gibco, USA) at 4°C. Fully isolated follicles were recovered by a micropipette.

The obtained follicles were randomly distributed into four groups: group I: fresh follicles; group II: follicles treated with slow-rate freezing; group III: follicles treated with OPS; and group IV: follicles treated with SSV.

### Slow-rate freezing and thawing

The slow-rate freezing and thawing procedure was performed according to the method described by Stachecki et al. [11]. Briefly, the follicles were placed in a basal freezing medium containing 1.5 mol/L dimethyl sulfoxide (DMSO) in PBS for 10 min, then follicles were transferred to a freezing medium containing 1.5 mol/L DMSO and 0.2 mol/L sucrose in PBS for 10-15 min. Two to five follicles were loaded into 0.25 mL French straws (Cryo Bio System, Paris, France). Subsequently, straws were placed in a programmable freezer (PLANER KRYO 10, UK), which was originally set at 25°C. The cooling rate was set at -2°C/min. At -7°C, we performed seeding by touching the side of each straw with forceps. Straws were held at -7°C for 10 min and cooled to -33°C with a rate of -0.3°C/min before immersion into LN_2_. Cryopreserved follicles were stored at -196°C.

For thawing straws were warmed in air at room temperature for 30 sec and then immersed in a water bath at 30°C for 40 sec. The content was expelled in a solution containing 1.0 M DMSO and 0.3 M sucrose, and follicles were equilibrated for 5 min. Then, follicles were transferred into the solution of 0.5 M DMSO and 0.3 M sucrose for 5 min followed by a solution of 0.3 M sucrose for 10 min and a final step using PBS for 20 min (10 min at room temperature and 10 min at 37°C).

### OPS and warming

The OPS and warming procedure was modified according to Maria Teresa Paramio et al [12]. Briefly, follicles were initially exposed to the first vitrification solution (4% ethylene glycol, EG in DPBS + 10% FBS) for 15 min. Subsequently, they were rinsed three times in the second vitrification solution (40% EG and 0.5 M sucrose in DPBS + 10% FBS) and equilibrated at room temperature for 20-30 sec. Then follicles were loaded in OPS straws (2-4 follicles per straw) and immediately plunged into LN_2 _for storage.

For warming straws were hold in air at room temperature for 3 sec and then the narrow end of the straw was directly immersed into the solutions. Rehydration and elution of cryoprotectants consisted of immersions in 0.5 M sucrose, 0.25 M sucrose, 0.125 M sucrose and basal medium (DPBS and 10% FBS), which were performed at room temperature for 2 min, respectively.

### SSV and warming

The SSV and warming procedure was modified from the method described by Lin et al. [13]. Briefly, follicles were initially exposed to the first vitrification solution (4% EG in DPBS + 10% FBS) for 15 min. Subsequently, they were rinsed three times in the second vitrification solution (35% EG and 0.5 M sucrose in DPBS + 10% FBS) and equilibrated at room temperature for 20-30 sec. Then follicles were directly dropped onto the surface of a metal plate, which was pre-cooled to approximately -150°C to -180°C by partial immersion in LN_2_. Droplets (in size of 6 μL) containing the vitrification solution and follicles were instantaneously vitrified into transparent spherical droplets. Droplets were transferred into 1.8 mL cryovials (Hamptom Research, USA) using LN_2_-cooled forceps and stored in LN_2_.

Warming was achieved by direct transfer into warming solution containing 0.25 M sucrose and 10% FBS in DPBS at 37°C for 5-10 min followed by three washes in DPBS.

### Follicle viability assessment

After warming, follicle viability was assessed according to the live-dead assay proposed by Cortvrindt et al. [14]. All the follicles, including fresh and thawed follicles, were incubated in DPBS containing 2 μM calcein-AM and 5 μM ethidium homodimer-I (Molecular Probes, Leyden, the Netherlands) in the dark at 37°C for 30 min. Follicles were subsequently washed in DPBS and observed under an inverted fluorescence microscope (Leica DMIRE 2, Germany) using appropriate filters. Green fluorescence was characteristic for live cells and red fluorescence for dead cells. Follicles were classified as follows: (i) 'totally viable' when the oocyte and all granulosa cells were intact; (ii) 'damaged' when the oocyte was alive but damage was observed in granulosa cell; and (iii) 'dead' when the oocyte and/or all granulosa cells were dead [15].

### Preparation of follicles for transmission electron microscopy

In our study, 65 follicles were examined for ultrastructural observation, and they were randomly selected from thawed follicles. Follicles were fixed in 2.5% glutaraldehyde solution overnight at 4°C, and then embedded in 2% gelose (Sigma). Subsequently, follicles were postfixed in 1% osmium tetroxide for 1 hr at 4°C, and washed three times in 0.1 M cacodylate buffer for 30 min. The samples were then dehydrated and embedded in Epoxy Resin. Finally, 60-80 nm ultra-thin sections were cut and examined on a transmission electron microscope [16]. The following selected organelles and structures were examined: mitochondria, rough endoplasmic reticulum, lipid droplet, vesicles, lysosomes, perivitelline space, microvilli, nucleus and oolemma. We recorded the ultrastructural modifications one by one and then calculated the percentages respectively.

### *In vitro *culture of isolated follicles

The *in vitro *culture protocol was modified from the method described by Amorim et al. [17]. Morphologically normal follicles with centrally located spherical oocytes were used for further *in vitro *culture. The isolated and thawed follicles were transferred into 1.5% of sodium alginate solution. Beads were formed by dropping two follicles into 0.25 M CaCl_2 _solution. The beads were transferred into five-well multidish for further culture. The culture medium consisted of MEM-Alpha supplemented with 10% FBS, 50 IU/mL penicillin (Sigma) and 50 mg/mL streptomycin sulfate (Sigma), 10 mg/mL insulin (Sigma), 5.5 mg/mL transferrin (Sigma), 6.7 ng/mL sodium selenite (Sigma) and 1 IU/mL recombinant Follicle Stimulating Hormone (R-FSH, Serono, Geneva, Switzerland) [18]. Following 72 hr of *in vitro *culture, the follicle diameter was measured. The spent medium was collected (500 μL per collection) for hormone assays. The estradiol level was measured by chemiluminescence with an ACS180-SE autoanalyzer (Bayer Diagnostics, Fernwald, Germany).

### Statistical analysis

Comparisons between percentages of viable follicles and proportions of ultrastructural alterations in the four groups were analyzed by χ^2 ^test. Statistical analysis for follicle diameters and estradiol levels were performed by analysis of variance (one-way ANOVA) with post hoc testing. *P *< 0.05 was considered as statistically significant.

## Results

### Assessment of follicle viability after different freezing procedures

**Figure 1:**
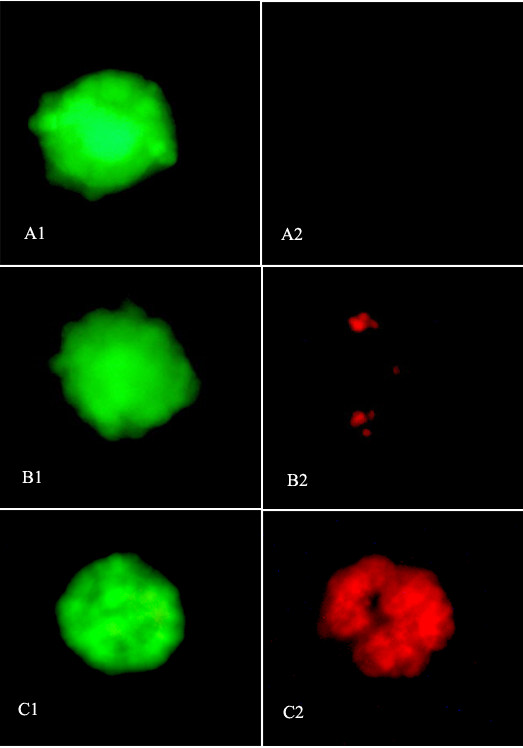
**Viability assessment of isolated follicles after cryopreservation**. Through the viability assessment, isolated follicles were classified into three categories. A, totally viable follicles: follicles with oocyte and all granulose cells viable (**1 **and **2 **showed the images obtained for the same follicle with two filters to visualize green fluorescence or red fluorescence for live or dead cells, respectively); B, damaged follicles: follicles with a viable oocyte and dead granulosa cells; and C, dead follicles: follicles with both oocyte and granulosa cells dead. Original magnification = 200×.

**Figure 2:**
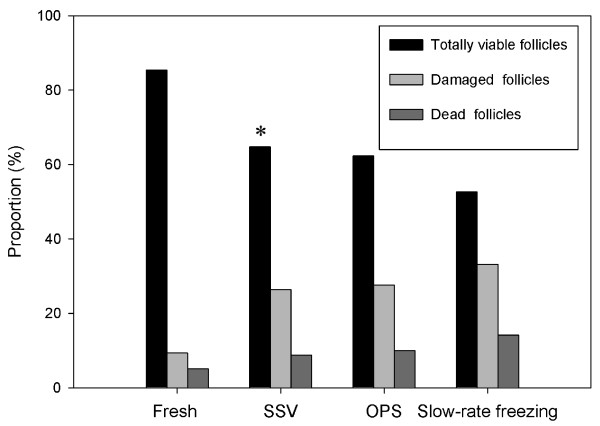
**Proportions of viable follicles after different freezing procedures**. The totally viability of follicles vitrified by SSV (64.76%) was slightly higher than that of the OPS group (62.38%) and significantly higher than that of the slow-rate freezing group (52.65%). *P < 0.05 versus the slow-rate freezing group.

876 follicles were subjected to viability assessment by calcein-AM and ethidium homodimer-I. The follicle numbers in the fresh, SSV, OPS and slow-rate freezing groups were 213, 227, 210 and 226, respectively. Fig. 1 shows follicles which were classified as 'totally viable', 'damaged' and 'dead', respectively. As shown in Fig. 2, the totally viable rate of follicles vitrified by SSV (64.76%) was slightly higher than that of the OPS group (62.38%) and significantly higher than that of the slow-rate freezing group (52.65%; *P *< 0.05).

### Ultrastructural observation

65 follicles were examined for ultrastructural morphology of mitochondria, rough endoplasmic reticulum, lipid droplet, vesicles, lysosomes, perivitelline space, microvilli, nucleus and oolemma. The follicle numbers in the fresh, SSV, OPS and slow-rate freezing groups were 12, 15, 18 and 20, respectively.

**Figure 3:**
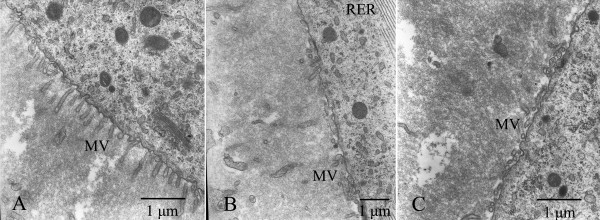
**Electron micrographs of microvilli (MV)**. A: Most microvilli (MV) were regularly arrayed. B: Microvilli abundance was reduced, and the shape of most microvilli (MV) was abnormal after treated with OPS. C: Microvilli abundance was reduced, and the shape of most microvilli (MV) was abnormal after treated with slow-rate freezing.

**Figure 4:**
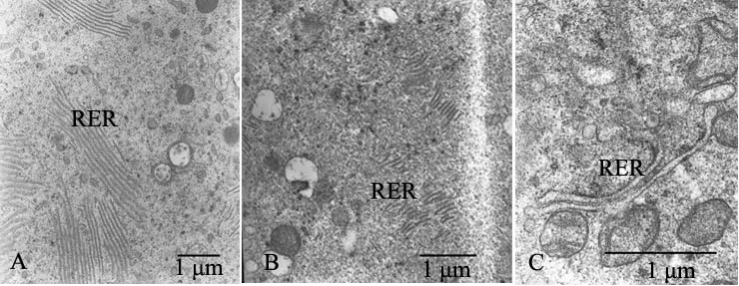
**Electron micrographs of rough endoplasmic reticulum (RER)**. A: The number of rough endoplasmic reticulum (RER) was large, and RER was well shaped. B: Most of RER were shorter after treated with OPS. C: RER was swollen, and a reduction of RER abundance was noted after treated with SSV.

**Figure 5:**
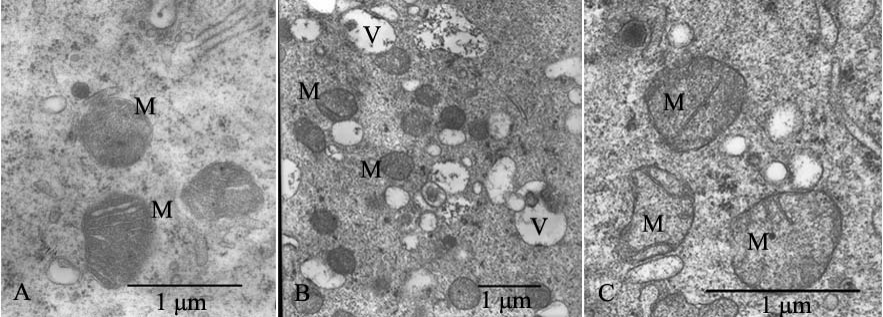
**Electron micrographs of mitochondria (M)**. A: Mitochondria (M) had normal cristae and electron-dense matrix. B: Accumulation of vesicles (V) was observed after treated with slow-rate freezing. C: Most mitochondria (M) were swollen and showed few or no cristae after treated with SSV.

**Figure 6:**
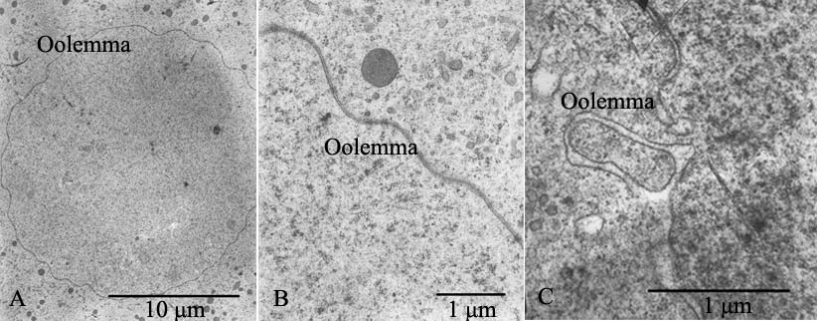
**Electron micrographs of Oolemma**. A, B: Oolemma was intact. C: Breakage of oolemma was noted after treated with slow-rate freezing.

In fresh follicles, the oolemma was intact and numerous microvilli projected into the perivitelline space (Fig. 3A). The amount of rough endoplasmic reticulum was abundant (Fig. 4A). Most mitochondria had normal cristae and electron-dense matrix (Fig. 5A), and only 1 of 12 fresh follicles displayed changes in mitochondria. There were almost no lipid droplets, vesicles or lysosomes found in these follicles. As shown in Fig. 6A, the oolemma was well-defined and intact.

**Figure 7:**
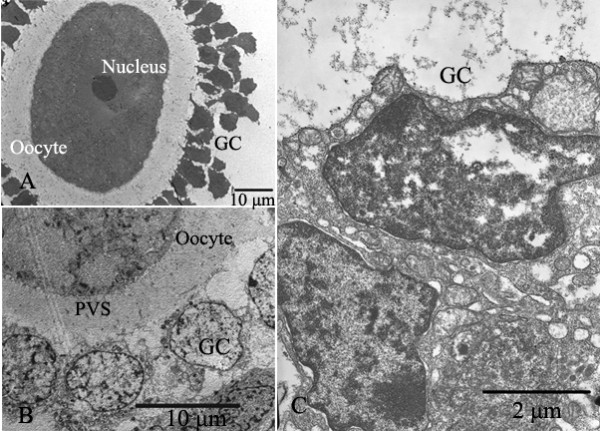
**Electron micrographs of granulosa cells (GC)**. A, B: The integrity of follicles was well preserved. Nucleus, oocyte and granulosa cells (GC) were indicated. C: Nucleus of granulosa cells (GC) was dissolved after treated with slow-rate freezing.

**Figure 8:**
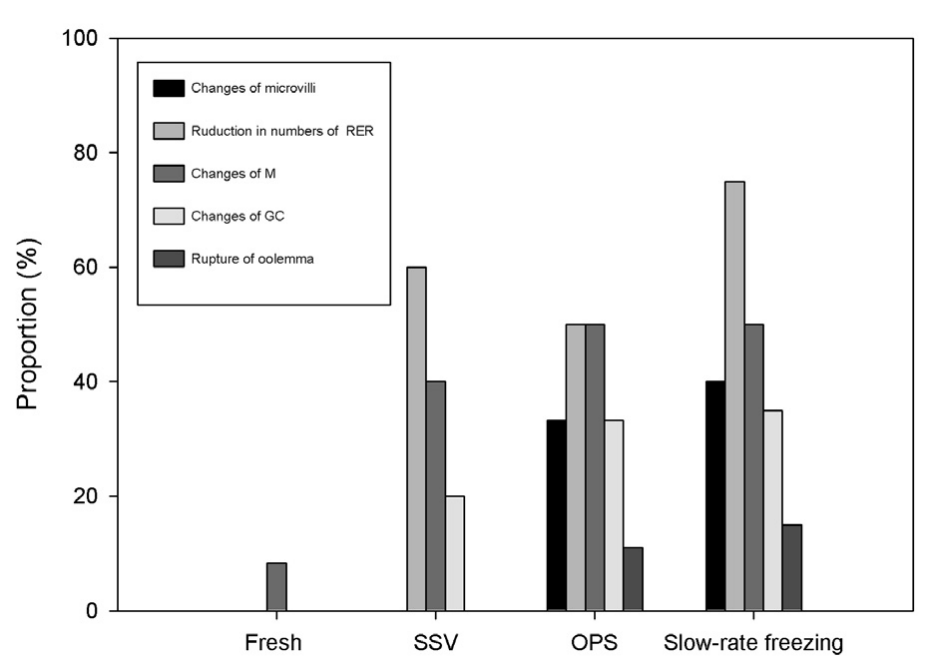
**Proportions of ultrastructural alterations after different freezing procedures**. The proportions of alterations (change of microvilli, change of mitochondria cristae, change of GC and rupture of oolemma) in follicles vitrified by SSV showed a decreasing tendency compared with those of follicles vitrified by OPS and frozen by slow-rate freezing. (M: Mitochondria, RER: rough endoplasmic reticulum).

Compared to fresh follicles we found the following alterations in follicles treated by SSV, OPS and slow-rate freezing: (1) the abundance of microvilli was reduced; (2) an abundance reduction of rough endoplasmic reticulum could be found in some cases; (3) some mitochondria were swollen, with few or no crista; (4) changes of GC; and (5) rupture of oolemma (Fig. 3, Fig. 4, Fig. 5, Fig. 6, Fig. 7). Overall the proportion of alterations (change of microvilli, change of mitochondria cristae, change of GC and rupture of oolemma) was relatively less in follicles vitrified by SSV compared to follicles vitrified by OPS and frozen by slow-rate freezing. Fig. 8 shows the proportions of reduction in the number of rough endoplasmic reticulum (SSV: 60.0%, OPS: 50.0% and slow-rate freezing: 75.0%) and changes of mitochondria cristae (SSV: 40%, OPS: 50.0% and slow-rate freezing: 50.0%). They both showed relatively higher than other types of alterations.

### Growth of thawed follicles *in vitro*

**Figure 9:**
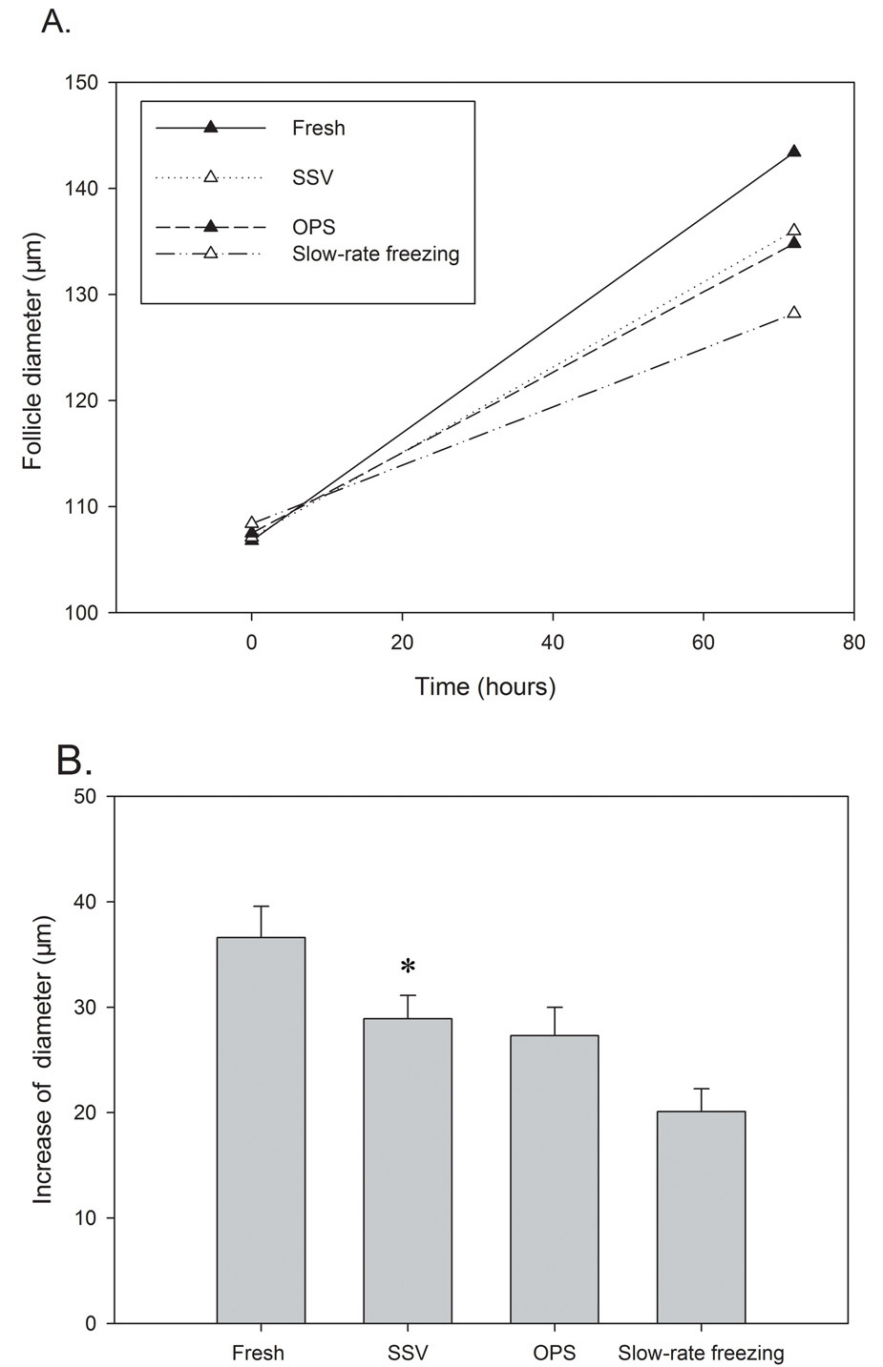
**Growth of thawed follicles cultured over a 72 hour culture period**. (A) Diameter of follicles cultured over 72 hr. Each line represents the follicle diameter measured before and after 72 hr of *in vitro *culture (N = 50 follicles per group). Data points are average diameters. (B) Increase of follicle diameter after *in vitro *culture. The increase in the follicle diameter of SSV group was significantly higher than that of the slow-rate freezing group. Columns are mean ± S.D.*P < 0.05 versus the slow-rate freezing group.

We compared follicular growth and changes in follicular diameter in a total of 200 follicles (50 follicles in each group) after *in vitro *culture. Fig. 9 shows the average follicle diameters measured at the beginning and at the end of the 72-hr culture period. As expected, fresh follicles exhibited maximal growth (increase in diameter: 36.60 ± 2.98 μm), while follicles of the slow-rate freezing group showed the minimal increase in diameter (20.10 ± 2.18 μm). In the SSV group we observed an increase of 28.90 ± 2.21 μm during the 72-hr culture period and this was significantly higher compared to that of the slow-rate freezing group (*P *< 0.05).

**Figure 10:**
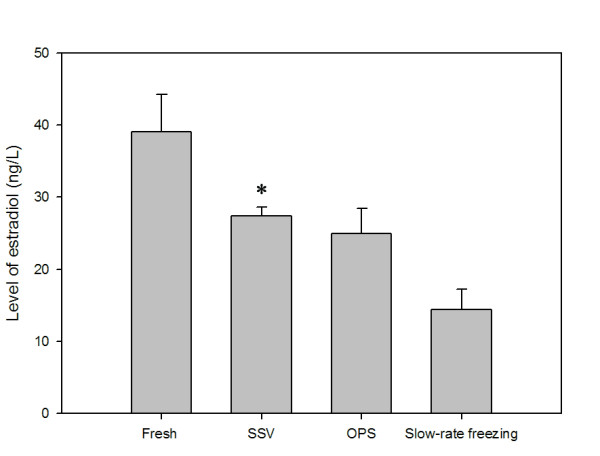
**Estradiol production in different groups after *in vitro *culture**. The estradiol level in the SSV group was significantly higher than those in the OPS and slow-rate freezing groups. Columns are mean ± S.D. *P < 0.05 versus the OPS and slow-rate freezing groups.

The levels of estradiol were also measured at the end of 72-hr culture period in our study. As shown in Fig. 10, the estradiol level, secreted from follicles in SSV group, was significantly higher than those in the OPS and slow-rate freezing groups (*P *< 0.05).

## Discussion

Isolation of follicles for subsequent cryopreservation is a relatively new approach for fertility preservation. Recently, Dolmans et al. developed a new enzymatic digestion protocol using Liberase. Liberase is a mixture of highly purified enzymes [19]. Previous studies have demonstrated that Liberase is a promising alternative to the rather impure collagenase preparations and allows for reproducible isolation of intact primordial and primary follicles from human ovarian tissues [17]. However, damage can also be found in follicles after enzymatic digestion with Liberase. In our study, 85.45% of follicles were totally viable after enzymatic isolation by Liberase, which is consistent with the results of other studies.

Isolated follicles can be cryopreserved by slow-freezing or by vitrification. Vitrification is a method with a high-concentration cryoprotectant combined with a fast cooling rate, which avoids ice crystal formation during the cryopreservation process [20]. The recent advance of ultrarapid vitrification procedures has been attributed to new devices which enabled high cooling rates, such as electron microscopic copper grids [21], OPS [22], cryoloop [23], microdrops [24], SSV [9] and nylon mesh [25]. Vitrification is a simple and economical method that can avoid potential risk of cellular injury caused by the formation of intracellular ice. However, exposure to high concentration of cryoprotectants is mandatory during vitrification and may result in chemical toxicity and osmotic damage to follicles.

In this study, we compared the efficiency and toxicity of the three cryopreservation methods (SSV, OPS and slow-rate freezing). Our results demonstrate that the rate of totally viable follicles of the SSV group (64.76%) was slightly higher than that of the OPS group (62.38%) and significantly higher than that of the slow-rate freezing group (52.65%; *P *< 0.05). Moreover, after *in vitro *culture, the increase of follicle diameter of SSV group was significantly higher than that of the slow-rate freezing group (*P *< 0.05). Therefore, our results suggest that the SSV technique results in high survival rates of follicles and retains their developmental capacity after cryopreservation and *in vitro *culture. These results could be attributed to several factors. First, the SSV method achieves a high cooling rate by using a combination of microdrops and improved heat exchange by direct contact with dry metal surface cooled by LN_2_. Rapid cooling is crucial for successful cryopreservation of follicles. The smaller sample size allows an increase of cooling and warming rates, which can reduce the toxicity of the cryoprotectant solution and chilling damages. These survival rates represent an improvement over the rates observed using other vitrification methods such as OPS, and can be attributed to the extremely fast cooling and warming rates achieved using the SSV system. In the SSV method, faster cooling is achieved due to the higher heat transfer rate on the cold, dry metal surface compared with the OPS technique, while liquid nitrogen boiling around the straw is the cooling interface in the OPS method. Using the SSV method for increasing the cooling rate between 20°C to -10°C, the "boiling off" phenomenon was disappeared[13]. These may also account for the decrease in the survival and developmental rates of follicles vitrified by the OPS method. Second, the thawing procedure of follicles vitrified by the SSV method is equally fast. The warming steps were performed according to the method described by Lin et al[13]. In this modified SSV method the warming steps were two steps instead of four steps, so the microdrops containing the follicles are directly dropped into a warming solution which results in a high warming rate. The extremely small volume also helps achieve a faster warming rate, thereby avoiding ice crystal formation during warming. This technical difference may also explain the decreased survival rates which we observed in follicles cryopreserved by the OPS and by the slow-rate freezing methods [26]. Last, small sample size in the SSV method reduces the occurrence of cracking. The volume of solution used in the slow-rate freezing method is greater than the microdrops in SSV method [16].

Moreover, concentrations of cryoprotectant may also be a factor affecting the survival rates of follicles. Chemical toxicity and osmotic damage to the follicles may result from exposure to the high concentration of cryoprotectants required for vitrification. A major concern about vitrification has been the use of higher concentrations of cryoprotectant in the suspending solution. Exposure to higher concentrations of cryoprotectant for longer periods may damage the follicles through toxicity of the cryoprotectant. Relatively low concentrations of cryoprotectant were required in the slow-rate freezing method. However, in our study, the rate of totally viable follicles of the SSV group was significantly higher than that of the slow-rate freezing group. Therefore, rapid cooling and thawing rates appear to be more crucial for successful follicle cryopreservation and the toxicity of cryoprotectants can be minimized by increasing the cooling rate[21].

The SSV method is more convenient for isolated follicle cryopreservation, because one microdrop can contain up to or even more than 10 follicles. Therefore, hundreds of follicles can be vitrified in a short time. Furthermore, follicles are not directly exposed to LN_2 _in the SSV method, which may help to avoid contamination by bacteria and/or viruses. This makes SSV a safe and reliable method for follicle cryopreservation. However, SSV method is also a challenging method, since the drop size on the cold surface must be carefully controlled in order to be less than 6 μL. Moreover, careful handling is mandatory in order to avoid that follicles do stick to the pipette or will be lost due to random drop dispersion caused by flipping movement [5].

In 2008, Lin et al cryopreseved mouse follicles using the SSV method[13]. In their study, they found that increasing the droplet size from 2 μL to 6 μL did not affect the finding of vitrificating formation of transparent spherical droplets and obtained similarly high survival rates in both groups. So we used the 6 μL volume in our study, and obtained high survival rate as well. High survival rates (91%) of vitrified-thawed preantral follicles isolated from two-week-old mice ovaries were obtained in that study, which was higher than those in our study. Differences in the method for viability assessment and the criteria of viable follicles in the two studies may have affected the observed survival rates.

In our study, ultrastructural examination after warming revealed a reduction of the abundance of microvilli and of rough endoplasmic reticulum as well as swollen mitochondria and ruptured oolemma in some cases. The proportions of reduction in the number of rough endoplasmic reticulum and in changes of mitochondria cristae were relatively higher than those of other types of alterations. The alterations of mitochondria and the reduction of rough endoplasmic reticulum abundance are most likely due to the change of osmotic pressure and the presence of free radicals during cooling and warming. Moreover, the toxicity of cryoprotectants and free radicals can induce the presence of small membrane-bound vesicles and lysosomes.

Our study demonstrates that follicles can survive following enzymatic isolation and cryopreservation. Furthermore, after *in vitro *culture using alginate hydrogels, most follicles showed an increase in follicle diameter. *In vitro *culture systems can generally be categorized into two approaches [27]. The first approach is an attached follicle approach, in which isolated follicles are placed on a two-dimensional surface and are permitted to attach and spread on the surface. The second approach is a three dimensional (3D) approach, in which follicles do not adhere to a surface and can maintain their native architecture. The 3D approach maintains cell-cell and cell-matrix interactions that are considered as critical regulators of follicle development [27]. In this respect, alginate has been proven as a suitable matrix, because it is firmer than collagen gel, and gives a better support for the growth of isolated follicles. Recently, West et al. has successfully applied a 3D system using alginate hydrogels to *in vitro *culture of isolated human follicles [28]. Furthermore, our study showed that this matrix was suitable for *in vitro *culture of isolated follicles due to its gentle gelling properties and biochemical characteristics, which was consistent with the results reported by Donnez et al [17].

## Conclusions

Taken together, our study demonstrates that cryopreservation of isolated rat follicles with the novel modified SSV method results in higher viability, less ultrastructural alterations, higher increase in follicle diameter and higher level of estradiol after *in vitro *culture. Compared with the conventional slow-rate freezing method and OPS method, the SSV method we presented is promising, more convenient and less expensive.

## Competing interests

The authors declare that they have no competing interests.

## Authors' contributions

WX conceived of the study, carried out follicle isolations, three different freezing procedures, follicle viability assessment, preparation of follicles for transmission electron microscopy and *in vitro *culture of isolated follicles, performed statistical analysis and drafted the manuscript. CZ participated in the design and coordination of the study and helped to draft the manuscript. JB participated in follicle isolations and preparation of follicles for transmission electron microscopy. MM was involved in drafting and finalizing the manuscript. YX, YL and TL participated in the design of the study. All authors read and approved the final manuscript.
